# High-Value-Added Compound Recovery with High-Temperature Hydrothermal Treatment and Steam Explosion, and Subsequent Biomethanization of Residual Strawberry Extrudate

**DOI:** 10.3390/foods9081082

**Published:** 2020-08-08

**Authors:** Juan Cubero-Cardoso, Ángeles Trujillo-Reyes, Antonio Serrano, Guillermo Rodríguez-Gutiérrez, Rafael Borja, Fernando G. Fermoso

**Affiliations:** 1 Instituto de Grasa, Spanish National Research Council (CSIC), Ctra. de Utrera, km. 1, 41013 Seville, Spain; juan.cubero@ig.csic.es (J.C.-C.); atrujillo@ig.csic.es (Á.T.-R.); antonio.serrano@ig.csic.es (A.S.); guirogu@ig.csic.es (G.R.-G.); rborja@cica.es (R.B.); 2 School of Civil Engineering, The University of Queensland, Campus St. Lucia-AEB Ed 49, 4067 St Lucia, QLD, Australia

**Keywords:** mesophilic anaerobic digestion, hydrothermal treatments, valorization, strawberry extrudate, phenols

## Abstract

This study was on the comparison of hydrothermal treatments at 170 °C (steam injection) and 220 °C (steam explosion) to solubilize the organic matter contained in residual strawberry extrudate, focusing on phenolic compounds that were susceptible to be extracted and on sugars. After the extraction step, the remaining strawberry extrudate phases were subjected to anaerobic digestion to generate biogas that would compensate the energy requirements of the suggested hydrothermal treatments and to stabilize the remaining waste. Hydrothermal treatment at 220 °C allowed the recovery of 2053 mg of gallic acid eq. per kg of residual strawberry extrudate. By contrast, after hydrothermal treatment at 170 °C, only 394 mg of gallic acid eq. per kg of residual strawberry extrudate was recovered. Anaerobic digestion processes were applied to the de-phenolized liquid phase and the solid phase together, which generated similar methane productions, i.e., around 430 mL CH_4_/g volatile solids, after both 170 °C and 220 °C hydrothermal treatments. Considering the latest observation, hydrothermal treatment at 220 °C is a preferable option for the valorization of residual strawberry extrudate (RSE) due to the high solubilization of valuable phenolic compounds that can be recovered.

## 1. Introduction

The strawberry sector has seen exponential growth in recent years, reaching 8.3 million tonnes in 2018 [1]. The strawberry sector includes not only a direct marketing of fresh fruit but also obtaining by-products derived from strawberries, such as yogurts, juices, or jams [2]. These by-products of strawberries generate a waste called residual strawberry extrudate (RSE) that is currently dumped in landfill. RSE is formed by achenes, fibers, and part of the juice of strawberries that is retained and rejected during the extrusion of the fruits to obtain a strawberry concentrate. This waste as well as the strawberry contains a wide variety of interesting bioactive compounds, with high concentrations of nutrients and phytochemicals, which could be recovered [3,4]. The great variety of nutrients and phytochemicals that strawberry contains is of great interest in our diet because they are beneficial to avoid or prevent different cardiovascular, neurological, cancerous, and other diseases [4,5].

The recovery of high-value-added compounds would allow considering the RSE as a by-product to be valorized instead of a waste to be treated. Different methods have been proposed to extract high-value-added compounds from strawberry, such as high-hydrostatic-pressure extraction [6], microwave hydrodiffusion and gravity [7], pulsed electric field [8,9], solvents [10], and hydrothermal treatments [11]. Hydrothermal treatments can be an attractive alternative to extract high-value-added compounds from the RSE because the control of the operation conditions (mainly temperature, pressure, and time) allow different solubilization grades to be obtained in the treated substrate. Hydrothermal treatments at medium temperatures (150–180 °C) solubilize most of the hemicellulose in the lignocellulosic biomasses but have a more limited effect over lignin and cellulose [12,13]. Alternatively, hydrothermal extraction at high temperatures and pressures (0.69–4.83 MPa) followed by a rapid decompression, so-called steam-explosion treatments, enhance the solubilization of cellulose with respect to the medium-temperature hydrothermal treatments, whereas the lignin can be also partially affected [13]. Furthermore, under steam-explosion conditions, the rupture of the hemicellulose–lignin bonds makes the treated substrate more accessible to the action of enzymes in a further biological process and, hence, more biodegradable [14]. This alternative would be very interesting to make accessible the compounds in both the RSE fibers and in the achenes, which are more resistant than the fiber.

Hydrothermal treatments allow the separation of the hydrothermally treated substrates into a solid and liquid phase, facilitating the recovery of valuable compounds. Most of the soluble valuable compounds are displaced to the generated liquid phases, facilitating their subsequent recovery. In addition, an adequate hydrothermal treatment could increase the concentration of valuable compounds to be extracted from the liquid phase, such as phenols or sugars, from the breakdown of the lignocellulosic fibers [15]. The recovery of phenolic compounds of the RSE would be of great relevance, not only from the environmental point of view but also due to their application in different sectors of the alimentary and pharmaceutical industries [16,17]. Previously, Rodriguez-Gutiérrez et al. [11] studied the effect of different hydrothermal treatments, i.e., at 90, 120, 150, and 170 °C during 60 min, and steam explosion at 200 °C (2 and 5 min), with and without acid addition, over strawberry extrudate. These authors observed a close positive relation in the hydrothermal treatments between the increment of the treatment temperature and the concentration of sugars and phenolic compounds. However, it is necessary to evaluate the effect of the hydrothermal temperature over the stability of the phenolic compounds, since some of the bioactive compounds in the strawberry have been described as thermosensitive [18,19]. The recovery of the phenolic compounds from the treated RSE allows the partial detoxification of the substrate, but a large amount of sugar-rich organic matter remains. This de-phenolized RSE must be correctly managed to avoid potential environmental risks associated with the uncontrolled putrefaction of the organic matter, such as greenhouse gas emissions, pollution of aquatic ecosystem due to lixiviates, etc. [20].

Anaerobic digestion is a valorization method that has been widely proposed for the stabilization of biodegradable substrates, which might be an interesting option to recover energy from RSE and to stabilize the organic matter [21]. The extraction of the phenolic compounds solubilized during the hydrothermal treatments would be beneficial for the subsequent anaerobic digestion process due to the inhibitory effect of these compounds over the anaerobic microorganisms, especially the methanogens [22,23]. Previous studies have demonstrated that a hydrothermal treatment at 150 °C allowed the extraction of 392 mg of gallic acid eq./kg RSE in relation to the phenols, and has been able to increase 19.1% of the methane potential compared to the untreated strawberry extrudate, showing that the biorefinery process proposed is a good option for handling this substrate [17]. Nevertheless, it must be borne in mind that the temperature increases can release soluble sugar-derived by-products such as furfural, 5-hydroxymethylfurfural (5-HMF), etc., which can be inhibitory for anaerobic digestion processes at certain concentrations [11,22].

The aim of this research was to compare the effect of hydrothermal treatments at 170 °C for 60 min and steam explosion at 220 °C for 5 min on the RSE characteristics. After the hydrothermal treatments and subsequent extraction of phenolic compounds, anaerobic digestion was carried out by means of BMP (biochemical methane potential) tests to study the effects of these hydrothermal pre-treatments on the biomethanization of untreated and thermally pre-treated strawberry extrudates.

## 2. Materials and Methods

### 2.1. Residual Strawberry Extrudate

The RSE was provided by Hudisa S.A, located in Huelva, Spain (37.281813, −7.239095, Huelva, Spain). Once collected, the RSE was immediately stored at −20 °C to avoid the uncontrolled fermentation of the substrate. 

### 2.2. Hydrothermal Treatment Systems and Separation of Liquid and Solid Phases

High-temperature hydrothermal treatments were carried out at temperatures of 170 and 220 °C. The first hydrothermal treatment was carried out at 170 °C and 5 kg/cm^2^ pressure for 60 min by means of a steam-treatment reactor by direct steam injection. The second treatment was carried out at 220 °C and 32 kg/cm^2^ pressure for 5 min, followed by a rapid decompression, in a pilot-scale steam-explosion reactor. Subsequently, the treated RSEs were centrifuged to separate the liquid phase (LP) and solid phase (SP). More details about the experimental equipment are included in the Appendix A (Appendix A.1).

### 2.3. Extraction of Phenolic Compounds

The extraction of the phenolic compounds from the LP was carried out by a chromatographic method using adsorbent resin Amberlite XAD-16 (more details in Appendix A, Appendix A.2). The phase obtained after extraction has been called the de-phenolized liquid phase (DLP). The compounds adsorbed on the resin were subsequently extracted with 200 mL EtOH 80% (*v*/*v*) and 40 mL EtOH 96%.

### 2.4. Anaerobic Digestion Experimental Procedure

The anaerobic digestibility of untreated residual strawberry extrudate and the different phases after hydrothermal treatments and sequence extraction of phenols were evaluated by biochemical methane potential (BMP) tests (more details about the experimental setup are included in the Appendix A, Appendix A.3). The BMP tests were carried out up to the total exhaust of the gas production (24 day period), which was monitored daily throughout the process.

### 2.5. Kinetic Study

The kinetic parameters for each experiment were determined numerically from the experimental data obtained by non-linear regression with the SigmaPlot Software (version 11.0) (Systat Software Inc., San Jose, California, USA). The kinetic model used was the logistic model (sigmoidal 4 parameters), which was previously described by Donoso-Bravo et al. [24]. A full description of the kinetic model can be found in the Appendix A, Appendix A.4).

### 2.6. Chemical Analyses

The following parameters were determined in the RSE and/or in the effluents of the reactors: total chemical oxygen demand (CODt), soluble chemical oxygen demand (CODs), total solids (TS), mineral solids (MS), total volatile solids (VS), and pH. All determinations were carried out in accordance with standard methods (American Public Health Association (APHA), 2017). The description of the analytical methods for the determination of total sugars, acid sugars, total phenols, and hydroxymethylfurfural (HMF) can be found in Trujillo-Reyes et al. [17]. The determination of the soluble compounds from the SP was carried out after a water extraction according to Thompson et al. [25] (more details are included in Appendix A, Appendix A.5).

## 3. Results and Discussion

### 3.1. Effect of Hydrothermal Treatments on the Substrate Characteristics

Table 1 summarizes the physicochemical characterization of the different phases obtained after carrying out the hydrothermal treatments, as well as the untreated RSE. Regardless of the hydrothermal treatment, VS accounted more than 90% of the TS in all the samples. The solubilization of organic matter through the hydrothermal treatments was evaluated by comparing the CODs of the sum of the LP and SP (expressed as mg O_2_/kg RSE) to the CODs in the untreated RSE. According to the results, hydrothermal treatments increased the CODs in comparison with untreated RSE up to 23% and 103%, at 170 and 220 °C, respectively (Table 1). Most of these soluble compounds were displaced to the LP after the hydrothermal treatments, which retained around 75% and 85% of the CODs, at 170 and 220 °C, respectively (Table 1). The increase in the solubilization of organic matter (CODs) and the decrease in VS indicates that the treatment at 220 °C is more severe than the treatment at 170 °C.

The conditions of the hydrothermal treatment had a high influence on the distribution of the total phenols between the SP and the LP. On one hand, after the hydrothermal treatment at 170 °C, total phenols were distributed at 57% in the SP and 43% in the LP (Table 1). It is worth noting that the sum of total phenols in LP and SP at 170 °C resulted in a loss of 8% of total phenols with respect to untreated RSE. On the other hand, at 220 °C, total phenols were distributed at 37% in the SP and 63% in the LP. On the contrary to treatment at 170 °C, total phenols increased around 180% with respect to untreated RSE after the hydrothermal treatment at 220 °C (Table 1). The decrease in the total phenol content at 170 °C could be explained by the thermosensitive character of some bioactive compounds in the strawberry, which are usually degraded at high temperatures [18,19]. At 220 °C, although some thermosensitive compounds would be also degraded, the steam-explosion pre-treatment is able to alter the cellulose and, even, partially the lignin [26], releasing phenolic compounds that were not available after less severe hydrothermal treatments [27,28].

The increase in the concentration of total sugars, i.e., considering the sum of LP and SP, was almost five times higher after the treatment at 170 °C and around six times higher after the treatment at 220 °C, with respect to untreated RSE (Table 1). Therefore, hydrothermal treatment at 220 °C allowed the solubilization of 37% more total sugars than at 170 °C (Table 1). On the contrary, the acid sugars decreased after the hydrothermal treatments in comparison to the untreated RSE, especially at 220 °C (Table 1). Other authors also reported a decrease in the concentration of acid sugars due to the application of high-temperature treatments [11,29]. As it was described for the phenolic compounds, the increase in the concentration of total sugars is explained by the degradation of the lignocellulosic fibers of the RSE during the hydrothermal treatments, especially at 220 °C [27,30].

HMF poses well-known inhibitory properties for microorganisms [31], which could limit the subsequent implementation of bioprocesses, such as anaerobic digestion [13,32]. HMF was not detected in the untreated RSE. After the hydrothermal treatments, the highest concentration of HMF was found at 170 °C, reaching a concentration of up to 2993 ± 29 mg/kg RSE in LP. On the contrary, hydrothermal treatment at 220 °C only resulted in a concentration of 155 ± 2 mg/kg RSE in LP despite the higher operational temperature compared to 170 °C. The formation of HMF is closely related with the application of high temperatures, which involves the conversion of carbohydrates to furans such as the HMF [11,26]. The higher concentration of HMF at 170 °C with respect to 220 °C can be explained by the longer treatment time used in the first treatment (170 °C) compared to that used in the steam-explosion treatment (220 °C), i.e., 60 and 5 min, respectively, since the formation of HMF has been reported to be time dependent [33].

### 3.2. Effect of the Extraction of Phenolic Compounds in the Liquid Phase

**Figure 1:**
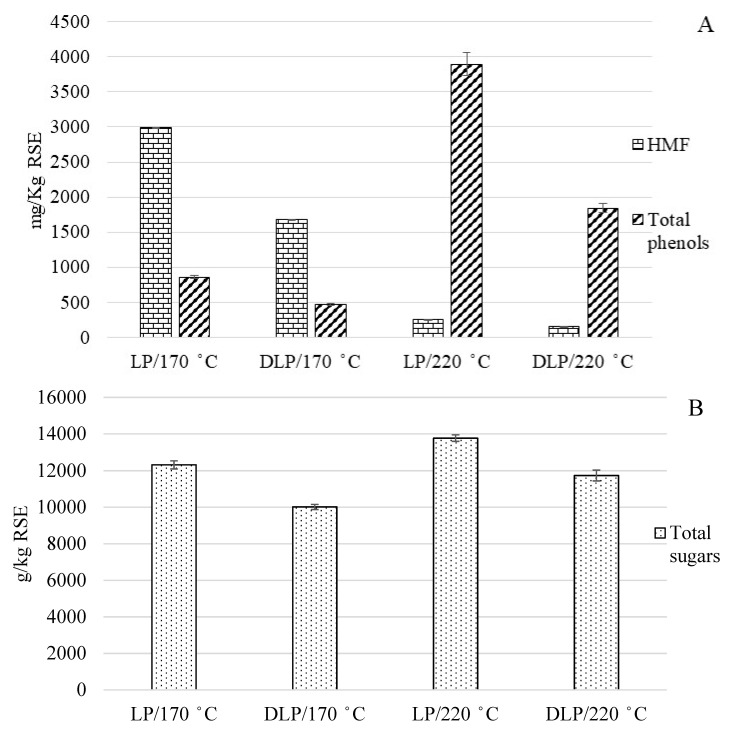
(**A**) Total phenols, hydroxymethylfurfural (HMF), and (**B**) total sugars from the liquid phase (LP) and the dephenolized liquid phase (DLP) after extraction of phenolic compounds with their standard deviations; where RSE, residual strawberry extrudate.

The efficiency of the Amberlite XAD-16 resin used for the extraction of phenolic compounds, i.e., total phenols and HMF, from LP is shown in Figure 1A. The extraction of total phenols presented an efficiency of up to 45% and 53% from the LP obtained at 170 and 220 °C, respectively (Figure 1A). These percentages were similar to those obtained with Amberlite XAD-16 resin for the extraction of phenolic compounds from other substrates such as mulberry and agricultural waste [34,35]. Amberlite XAD-16 resin was also able to remove up to 43% and 37% of HMF from the LP obtained at 170 and 220 °C, respectively (Figure 1A). Amberlite XAD-16 resin normally is used to extract hydrophilic and polar compounds, for this, it can be seen as having a good efficiency in the extraction of phenolic and HMF compounds [36]. However, the Amberlite XAD-16 resin did not affect to most of the total sugars in both LPs, retaining only around 18% and 15% of the total sugars at 170 and 220 °C, respectively (Figure 1B). 

### 3.3. Anaerobic Digestibility Study after the Application of Hydrothermal Treatments at High Temperatures and Subsequent Extraction of Phenolic Compounds

#### 3.3.1. Methane Potential and Kinetic Study of the Anaerobic Process after Treatment at 170 °C

A BMP test was carried out to evaluate the biomethane production from RSE, LP, SP, and DLP as well as the mixtures SP + LP and SP + DLP, after the hydrothermal treatment at 170 °C. Table 2 shows the results of the methane production, as well as the characterization of the effluents of the BMPs at the end of the digestion time. As can be seen, pH was very similar for all the conditions with values between 7.7 and 7.8 (Table 2), and, thereby, within the recommended range for an adequate methanogenic activity, i.e., 7.3–7.8 [37]. The optimal pH values at the end of the experimental time, despite of the acidic character of the substrates (Table 1), indicated a proper buffering of the anaerobic digestion systems (BMPs), which presented alkalinity concentration values in a range of 5000–7000 mg CaCO_3_/L.

Table 2 also shows that the amount of organic matter (VS and CODs) after the anaerobic digestion processes of the different substrates was in all cases less than 10 g VS/kg and 1.5 g O_2_/L, respectively. It also shows that the concentration of total phenols determined after the anaerobic digestion processes of the different substrates was in all cases less than 180 mg gallic acid eq./L, this concentration was much lower than those found as inhibitory in anaerobic digestion processes [38,39]. Finally, Table 2 also shows the biodegradability values, which were calculated from methane production. LP and DLP reached high degradation values, close to 100%, while the SP had the lowest biodegradability (67%). Co-digesting these phases, high biodegradability values of 81% for SP + LP and 90% for SP + DLP were achieved. It can be observed that after the extraction of phenolic compounds a 9% improvement in biodegradability was attained. 

**Figure 2:**
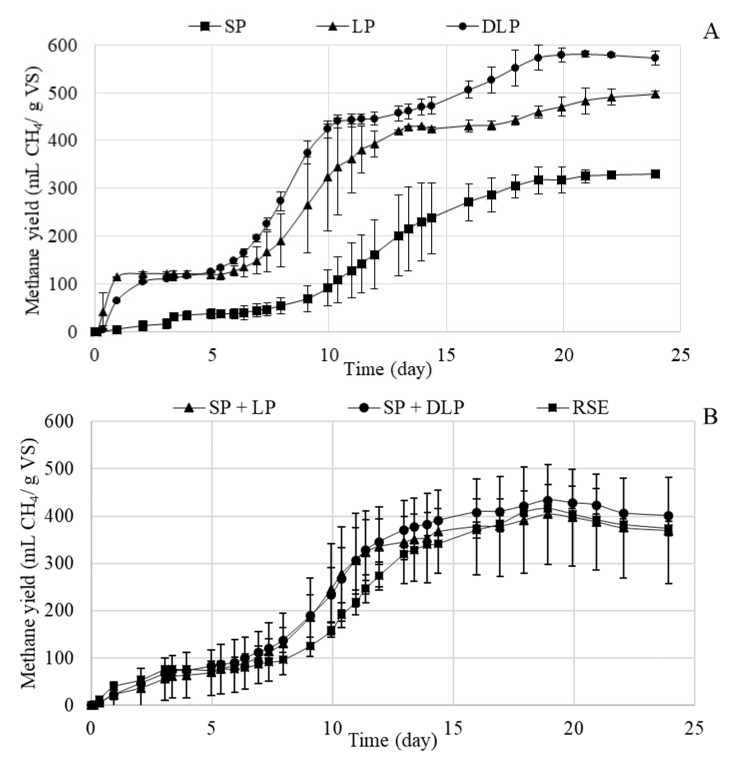
(**A**): Variation of cumulative methane production (mL CH_4_/g VS) (VS, total volatile solids) with time for LP (liquid phase), SP (solid phase), and DLP (dephenolized liquid phase) after 170 °C hydrothermal treatment with their standard deviations; (**B**): Variation of cumulative methane production (mL CH_4_/g VS) with time for the untreated RSE (residual strawberry extrudate), SP + LP and SP + DLP mixtures after 170 °C hydrothermal treatment with their standard deviations.

Figure 2A,B shows the graph of cumulative methane production (mL CH_4_/g VS) versus digestion time (days) for untreated RSE and for LP, SP, and DLP after 170 °C hydrothermal treatment as well as for the mixtures SP + LP and SP + DLP. Figure 2A,B shows that, at the beginning of the test, all samples had a small increase in methane production during the first 2 days. Although a slight difference was detected, since LP had a higher production in these initial two days compared to DLP, which could indicate that the extracted phenolic compounds were easily biodegradable (Figure 2A). The heterogeneity of the RSE, where some components such as the fibers present a slow degradation, whereas some soluble compounds are easily biomethanized, could be responsible for the stepped curve in Figure 2A [40]. Next, from day 2 to day 7, approximately, a lag phase (latency period or adaptation) was observed in the curves of all substrates. From day 7 onward, continuous exponential growth was observed until, approximately, day 15, when production began to be constant for all samples. LP and DLP were the substrates that produced the highest methane productions, while the SP was the fraction that generated the lowest methane production. In Figure 2A, a slight difference in the cumulative methane production between LP and DLP can be seen. The slightly higher cumulative methane production in DLP could be a consequence of the extraction process, mainly by the reduction of HMF with respect to the high concentration determined in LP [13,31]. The combination of the SP + LP and SP + DLP gave methane productions similar to that achieved for untreated RSE (Figure 2B), which indicates that produced HMF did not significantly affect the overall methane production.

Table 3 shows the values of the parameters obtained from the logistic model (sigmoidal 4 parameters) for the different substrates and mixtures studied after the hydrothermal treatment at 170 °C. The high values of R^2^ obtained, as well as the low values of errors and standard error of estimates (S.E.E.) indicated that the experimental data fit correctly to the proposed model. The maximum methane production rate, designated by *R_m_*, of the SP + LP mixture was 8.3% higher than the maximum production rate of untreated RSE (Table 3). The maximum methane production was reached for the mixture of SP + DLP, and the value of *R_m_* for this mixture was 5.5% higher than that for untreated RSE (Table 3). Therefore, it can be said that applying the 170 °C treatment and performing the anaerobic digestion process after the hydrothermal treatment and subsequent extraction of phenols, no significant change both in the maximum methane production and maximum methane production rate were observed, and these values were similar both in the RSE and in the mixture SP + DLP. Table 3 also shows the lag time data (ʎ) for all the substrates assayed. As can be seen, and similar to what occurred for both the maximum methane production and maximum methane rate, the lag values were very similar for RSE and for the mixture SP + DLP, showing the lowest value for DLP (8.3 days).

#### 3.3.2. Methane Potential and Kinetic Study of the Anaerobic Digestion Process after Hydrotreatment at 220 °C

BMP tests were carried out to evaluate the energy recovery from untreated RSE, and for LP, SP, and DLP after 220 °C hydrothermal treatment, as well as for the mixtures SP + LP and SP + DLP. Table 4 shows the values of the analytical parameters of the effluents obtained after the BMP tests for discussion of methane production, stability, and biodegradability. The stability of the anaerobic process was evaluated through pH and alkalinity values. The pH values, 7.6–7.8, remained within the recommended range for adequate methanogenic processes (from 7.3 to 7.8) [37] (Table 4). The alkalinity values after the BMP tests varied between 5000 and 6000 mg CaCO_3_/L, values high enough to buffer possible pH variations, as can be seen in the final pH values of the biomethanization processes. Table 4 shows that the amount of organic matter (VS and COD_S_) after the anaerobic digestion processes of the different substrates was in all cases less than 8.5 g VS/kg and 2 g O_2_/L, respectively. It also shows that the concentration of soluble phenols found after the anaerobic digestion processes of the different substrates was in all cases less than 201 mg gallic acid eq./L and these concentrations were much lower than those studied as inhibitory in anaerobic digestion processes [38,39]. Finally, Table 4 also shows that the biodegradability (calculated from methane production) of the LP and DLP reached values around 100%, while, on the contrary, the SP had the lowest biodegradability (45%). The co-digestion of these phases achieved biodegradability values of 84% for SP + LP and 78% for SP + DLP, these biodegradability values were very similar to that achieved for untreated RSE.

**Figure 3:**
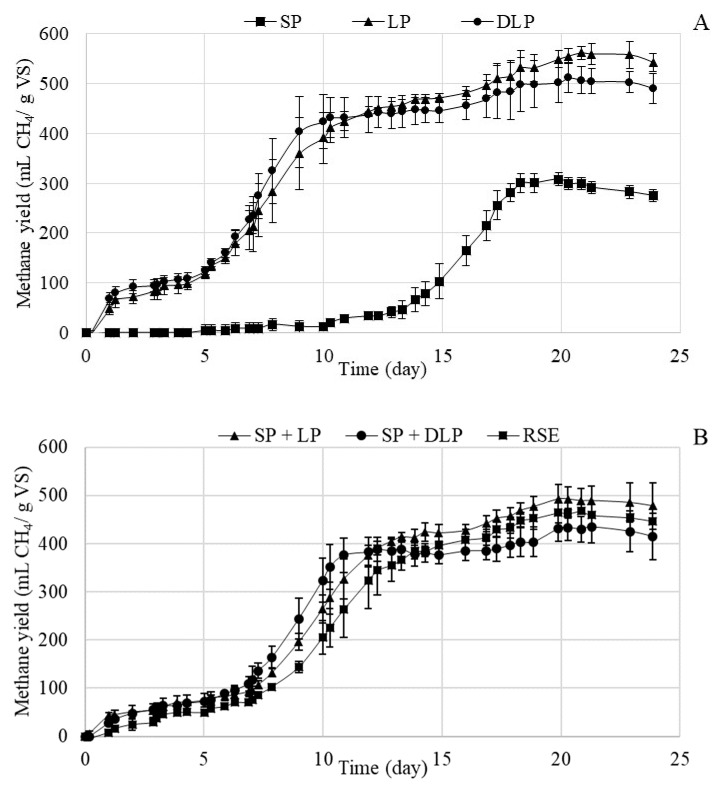
(**A**): Variation of cumulative methane production (mL CH_4_/g VS) (VS, total volatile solids) with time for LP (liquid phase), SP (solid phase), and DLP (dephenolized liquid phase) after 170 °C hydrothermal treatment with their standard deviations; (**B**): Variation of cumulative methane production (mL CH_4_/g VS) with time for the untreated RSE (residual strawberry extrudate), SP + LP and SP + DLP mixtures after 170 °C hydrothermal treatment with their standard deviations.

Figure 3A,B shows the variation of cumulative methane production (mL CH_4_/g VS) with digestion time (days) for untreated RSE and for the LP, SP, and DLP after 220 °C hydrothermal treatment, as well as for the mixtures SP + LP and SP + DLP. Figure 3 shows that at the beginning of the digestion time (2 days), except for the SP, a small increase in methane production was observed. Next, from day 2 to day 7, approximately, a lag phase (latency or adaptation period) was observed in the curves of all the substrates, except for the SP (Figure 3A), for which the lag phase starts at the beginning of the assay. From day 13 for the SP and day 7 for the other phases, a continuous exponential growth was observed until reaching day 15, where the production begins to be constant for all the samples, except for SP where it began on day 18. LP and DLP were the substrates that produced more methane and SP the one with the lowest methane production (Figure 3A). LP and DLP had similar cumulative methane production curves, despite the higher concentration of phenolic compounds in LP with respect to DLP. The same trend was also reported in the methane production of LP and DLP obtained after similar thermal treatment studies on olive-mill solid waste and raspberry extrudate [17,22]. The combinations of the SP + LP and SP + DLP gave methane productions similar to that of untreated RSE, as occurred in the anaerobic digestion of these mixtures from hydrothermal treatment at 170 °C. 

Table 5 shows the values of the parameters obtained from the logistic model (sigmoidal 4 parameters) for the different substrates and mixtures studied after hydrothermal treatment at 220 °C (steam explosion). As can be observed, the high values of R^2^ obtained, as well as the low values of errors and standard errors of estimate (S.E.E.) indicated that the experimental data fit correctly to the proposed model. The maximum methane production rate, *R_m_*, of the mixture SP + DLP was 43.9% higher than the *R_m_* value of the untreated RSE (Table 5). With respect to the maximum methane production, the mixture SP + DLP produced 19.34% less methane than that obtained for untreated RSE (Table 5). Absence of the extracted phenolic compounds improved production rate, however, the degradability of such compounds is also missed in the final methane production as seen in the slightly lower methane production value of SP + DLP compared to RSE and the mixture SP + LP (Table 5). Table 5 also shows the lag time data (ʎ) for each one of the substrates tested, this value was very similar for the RSE and for the mixture SP + DLP and SP + LP. 

## 4. Conclusions

The hydrothermal treatment at 220 °C for 5 min allowed obtaining a double concentration of soluble organic matter and up to three times more phenolic compounds than at 170 °C for 60 min. The hydrothermal treatment at 220 °C resulted in a much lower concentration of undesirable HMF, a known inhibitor of anaerobic microorganisms, in comparison to 170 °C for 60 min. Despite this, no marked differences were observed in the methane production between the SP + DLP mixtures of both treatments and untreated RSE, reaching a methane yield around 430 mL CH_4_/g volatile solids. Considering the latest observation, hydrothermal treatment at 220 °C for 5 min is a preferable option for the valorization of RSE due to the high solubilization of valuable phenolic compounds that can be recovered. The optimization of the hydrothermal treatment will facilitate the valorization of the RSE, which would be considered a source of benefits, i.e., phenolic compounds and energy, instead of a waste to be treated.

## Figures and Tables

**Table 1:** Physicochemical characterization of untreated RSE and different treated phases (mean values ± standard deviations; n.d.: non-detected).

		RSE	170 °C, 60 min, 5 kg/cm^2^		220 °C, 5 min, 32 kg/cm^2^	
		RSE	SP	LP	SP	LP
**pH**		3.7 ± 0.1	4.3 ± 0.1	3.8 ± 0.1	3.6 ± 0.1	3.8 ± 0.1
**TS**	(mg/kg RSE)	144,681 ± 3986	94,280 ± 871	32,044 ± 1024	58,092 ± 1268	47,120 ± 1515
**VS**	(mg/kg RSE)	139,336 ± 4423	90,801 ± 3405	29,430 ± 606	56,162 ± 2106	43,296 ± 891
**COD_t_**	(mg O_2_/kg RSE)	200,366 ± 6312	116,027 ± 1918	40,516 ± 373	89,775 ± 3220	63,910 ± 915
**COD_S_**	(mg O_2_/kg RSE)	47,237 ± 317	14,423 ± 463	43,927 ± 352	14,279 ± 316	82,027 ± 1194
**Total Phenols**	(mg gallic acid eq./kg RSE)	2185 ± 64	1141 ± 30	858 ± 0	2200 ± 3	3895 ± 165
**Total Sugars**	(mg glucose eq./kg RSE)	2023 ± 99	875 ± 23	12,322 ± 222	1644 ± 34	13,769 ± 178
**Acids Sugars**	(mg galacturonic acid eq./kg RSE)	6.28 ± 0.12	2.11 ± 0.06	2.93 ± 0.06	0.29 ± 0.00	0.38 ± 0.01
**HMF**	(mg/kg RSE)	n.d.	73 ± 1	2993 ± 29	5 ± 1	155 ± 2

RSE: residual strawberry extrudate, LP: liquid phase, SP: solid phase, TS: total solids, VS: volatile solids, COD_t_: total chemical oxygen demand, COD_s_: soluble chemical oxygen demand, HMF: hydroxymethylfurfural.

**Table 2:** Analytical characterization of effluents from the anaerobic digestion process at the end of the biochemical methane potential (BMP) tests (after treatment at 170 °C) with their standard deviations.

	RSE	SP	LP	DLP	SP + LP	SP + DLP
**pH**	7.7 ± 0.1	7.8 ± 0.1	7.8 ± 0.1	7.8 ± 0.1	7.7 ± 0.1	7.8 ± 0.1
**Alkalinity** **(mg CaCO_3_/L)**	5768 ± 278	5902 ± 47	6174 ± 145	6590 ± 145	6049 ±51	6028 ± 46
**TS (mg/kg)**	15,391 ± 442	15,570 ± 156	15,272 ± 118	15,057 ± 327	15,104 ± 265	15,610 ± 655
**VS (mg/kg)**	9918 ± 369	9961 ± 235	9250 ± 173	9226 ± 226	9837 ± 367	9981 ± 403
**COD_S_** **(mg O_2_/L)**	1181 ± 48	1184 ± 56	1448 ± 59	1464 ± 17	1224 ± 79	1196 ± 65
**Total phenols** **(mg gallic acid eq./L)**	158 ± 8	163 ± 1	172 ± 6	171 ± 9	159 ± 14	157 ± 4
**Experimental methane production** **(mL CH_4_/g VS)**	416 ± 8	329 ± 7	497 ± 6	580 ± 7	403 ± 105	434 ± 32
**Biodegradability (based on VS) (%)**	74	67	94	125	81	90

**Table 3:** Values of the parameters obtained from the logistic model (sigmoidal 4 parameters) for the different substrates and mixtures studied (hydrothermal treatment at 170 °C).

Substrates	*P* (mL CH_4_/g VS)	*R_m_* (mL CH_4_/g VS·d)	ʎ (d)	*B*_0_ (mL CH_4_/g VS)	R^2^	Error (%)	S.E.E.
**RSE**	345 ± 6	50.6 ± 4.4	11.45 ± 0.09	66 ± 4	0.9964	1.2	8.88
**SP**	305 ± 4	35.6 ± 1.5	12.37 ± 0.07	22 ± 3	0.9987	0.4	4.34
**LP**	351 ± 10	59.8 ± 9.3	9.5 ± 0.1	107 ± 8	0.9899	6.3	11.61
**DLP**	540 ± 54	52.4 ± 15.1	8.3 ± 0.5	22 ± 47	0.9755	3.0	10.9
**SP + LP**	341 ± 7	55.3 ± 5.6	9.5 ± 0.1	51 ± 6	0.9952	2.6	10.16
**SP + DLP**	364 ± 5	54.8 ± 4.1	10.02 ± 0.08	63 ± 4	0.9977	1.5	7.45

S.E.E.: standard error of estimates.

**Table 4:** Analytical characterization of effluents from the anaerobic digestion processes at the end of the BMP tests (after treatment at 220 °C) with their standard deviations.

	RSE	SP	LP	DLP	SP + LP	SP + DLP
**pH**	7.7 ± 0.1	7.8 ± 0.1	7.7 ± 0.1	7.7 ± 0.1	7.7 ± 0.1	7.6 ± 0.1
**Alkalinity (mg CaCO_3_/L)**	5617 ± 18	5328 ± 185	5482 ± 141	5333 ± 142	5328 ± 204	5264 ± 59
**TS (mg/Kg)**	12,987 ± 180	13,412 ± 667	12,533 ± 237	12,702 ± 211	13,082 ± 354	13,401 ± 290
**MS (mg/Kg)**	5478 ± 246	5062 ± 440	4988 ± 142	5343 ± 412	5331 ± 279	5276 ± 351
**VS (mg/Kg)**	7685 ± 256	8425 ± 180	7370 ± 307	7171 ± 276	7848 ± 345	7975 ± 572
**COD_S_ (mg O_2_/L)**	767 ± 33	1973 ± 89	1438 ± 39	1020 ± 62	1144 ± 63	937 ± 10
**Total phenols (mg gallic acid eq./L)**	134 ± 1	159 ± 2	201 ± 7	158 ± 9	176 ± 3	165 ± 5
**Theoretical methane** **production (mL CH_4_/g VS)**	559	661	564	481	590	554
**Experimental methane production (mL CH_4_/g VS)**	468± 4	299 ± 12	562 ± 13	512 ± 30	493 ± 30	434 ± 27
**Biodegradability (based on VS) (%)**	84	45	100	106	84	78

**Table 5:** Values of the parameters obtained from the logistic model (sigmoidal 4 parameters) for the different substrates and mixtures studied (hydrothermal treatment at 220 °C).

Substrates	*P* (mL CH_4_/g VS)	*R_m_* (mL CH_4_/g VS·d)	ʎ (d)	*B_0_* (mL CH_4_/g VS)	R^2^	Error (%)	S.E.E.
**RSE**	429 ± 9	54.7 ± 6.3	10.6 ± 0.1	27 ± 6	0.9998	2.2	11.56
**SP**	310 ± 4	62.3 ± 4.9	15.72 ± 0.07	6 ± 1	0.9962	2.8	7.92
**LP**	552 ± 34	49.3 ± 9.3	7.8 ± 0.3	8.3 ± 1.5	0.9886	0.1	10.92
**DLP**	412 ± 17	68.2 ± 14.3	7.4 ± 0.1	70 ± 14	0.9838	5.7	11.10
**SP + LP**	431 ± 8	60.2 ± 8.6	10.0 ± 0.1	44 ± 6	0.9942	3.3	12.43
**SP + DLP**	346 ± 8	78.7 ± 12.8	8.7 ± 0.1	59 ± 7	0.9893	6.5	10.31

## Appendix A

### Appendix A.1. Hydrothermal Treatment Systems and Separation of Liquid and Solid Phases

High-temperature hydrothermal treatments were carried out using a steam-treatment reactor with 100 L of capacity, which can reach temperatures up to 190 °C and a maximum pressure of 1.2 MPa. Heating of the strawberry extrudate was performed by direct steam injection. Wherein, 12.59 kg of RSE was introduced in the reactor at 170 °C and 5 kg/cm^2^ pressure for 60 min. After the treatment period, the sample was cooled to 50 °C and then centrifuged at 4700 g/1450 rpm (Comteifa, S. L., Barcelona, Spain) to separate the liquid phase (LP) and solid phase (SP). Steam-explosion treatments were performed in a pilot-scale reactor (Nusim, S.A., Madrid, Spain). The reactor was equipped with a stainless-steel deposit with 2 L of capacity. The steam-explosion reactor was loaded with 400 g of RSE, which was heated at a temperature of 220 °C and 32 kg/cm^2^ pressure for 5 min. An electronic computing device controlled the time and the temperature in a preprogrammed manner. After each treatment, the centrifuge (4226 Pacisa) was used to separate the liquid phase (LP) and solid phase (SP).

### Appendix A.2. Extraction of Phenolic Compounds

The extraction of the phenolic compounds was carried out with a column, 4.5 cm in diameter and 140 cm in height, filled with 100 mL of adsorbent resin Amberlite XAD-16. This Amberlite was dissolved in water corresponding to 12 cm in height in the column for the hydrothermal treatment of 170 °C, while in the hydrothermal treatment of 220 °C, a column 2 cm in diameter and 50 cm in height was used, filled with 25 mL of adsorbent resin Amberlite XAD-16 dissolved in water corresponding to 8 cm in height in the column. Amberlite XAD-16 is an adsorbent resin that retains a large percentage of phenols. These adsorption resins are highly crosslinked polymers with a large surface and numerous pores. In recent years, many studies have shown the efficacy of this type of resin in the design of new adsorption–desorption processes retaining a large number of bioactive compounds such as phenols [41]. Extractions were carried out from 2 L of liquid phase after hydrothermal treatment at 170 °C and from 0.5 L of liquid phase after hydrothermal treatment at 220 °C. The phase obtained after extraction has been called the de-phenolized liquid phase (DLP). The compounds adsorbed on the resin were extracted with 200 mL EtOH 80% (*v*/*v*) and 40 mL EtOH 96% according to Fernández-Bolaños et al. [42].

### Appendix A.3. Anaerobic Digestion—Experimental Procedure

The anaerobic digestibility of untreated residual strawberry extrudate and the different phases after hydrothermal treatments and sequence extraction of phenols were evaluated by biochemical methane potential (BMP) tests. BMP tests were carried out in 250 mL Erlenmeyer flasks using a working volume of 240 mL. In all cases, the BMP reactors were loaded with an inoculum/substrate ratio of 2:1, based on VS. All these assays were carried out in triplicate. BMP reactors were sealed, and the headspace of each flask was flushed with nitrogen at the beginning of the assay. All reactors were submerged in a bath of thermostatic water under mesophilic conditions (35 °C), and they were continuously stirred by magnetic bars to favor the transfer of matter between inoculum and substrate. The produced biogas was passed through a 2 N NaOH solution to capture CO_2_ and the remaining gas was assumed to be methane. The volume of methane was measured by liquid displacement. The BMP tests were carried out in the time interval required to exhaust gas production and VS removal (24 day period). Methane production was monitored daily throughout the process. A sludge from the anaerobic treatment of wastewater from the beer industry of “HEINEKEN SPAIN, S. A.,” (Seville, Spain) was used as an inoculum source. The main characteristics of the anaerobic inoculum were as follows: pH = 7.4 ± 0.1; alkalinity = 2500 ± 20 mg CaCO_3_/L; TS = 72,000 ± 200 mg/kg; MS = 14,000 ± 350 mg/kg; VS = 58,000 ± 400 mg/kg.

### Appendix A.4. Kinetic Study

The kinetic parameters and mathematical adjustment for each experiment were determined numerically from the experimental data obtained by non-linear regression, using the Software SigmaPlot (version 11.0). The kinetic model used was the logistic model (sigmoidal 4 parameters) (Equation (A1)):

B_2_ = B_0_ + P/[1 + exp⁡(−4 × R_m_ × (t − λ)/(P + 2))] (A1)

where B_2_ is the cumulative methane production during the second stage (mL CH_4_/g VS), B_0_ is the cumulative methane production at the beginning of the second stage (mL CH_4_/g VS), P is the maximum methane production obtained in the second stage (mL CH_4_/g VS), R_m_ is the maximum rate of methane production (mL CH_4_/g VS·d), t (d) is the time and *λ* (d) is the delay time.

In addition, R^2^, error (%), and standard error of estimate (S.E.E.) were determined to evaluate the fit and accuracy of the results. The error was defined as the difference in percentage between the final experimental cumulative methane production and the theoretical value predicted by the model.

### Appendix A.5. Chemical Analyses

To measure the water-soluble compounds of the RSE and of the solid phase (SP) after the hydrothermal treatments, an extraction was carried out by adding 160 g of distilled water to 20 g of sample and keeping it stirred for 24 h. After this time, the liquid was centrifuged and microfiltered with 0.45 μm nylon microfilters. This method allows to quantify the soluble compounds in the solid sample of this work and is also widely used for composting analysis [25]. To extract the soluble compounds from the liquid phase (LP) and de-phenolized liquid phase (DLP), it was centrifuged again to remove solid-phase residues in suspension and microfiltered with 0.45 μm nylon microfilters. The following parameters were determined in the RSE and/or in the effluents of the reactors: total chemical oxygen demand (COD_t_), soluble chemical oxygen demand (COD_s_), total solids (TS), mineral solids (MS), total volatile solids (VS), and pH. All determinations were carried out in accordance to standard methods [43].
